# Comparative Evaluation of Periapical Expulsion Using Manual, Rotary, and Reciprocating Instrumentation With EndoVac Irrigation: An In Vitro Study

**DOI:** 10.7759/cureus.77975

**Published:** 2025-01-25

**Authors:** Sachin Metkari, Sanpreet S Sachdev, Pravin Patil, Manoj Ramugade, Kishor D Sapkale, Kulvinder S Banga, Dinesh Rao

**Affiliations:** 1 Conservative Dentistry and Endodontics, Nair Hospital Dental College, Mumbai, IND; 2 Oral Pathology and Microbiology, Bharati Vidyapeeth (Deemed to be University) Dental College and Hospital, Navi Mumbai, IND; 3 Conservative Dentistry and Endodontics, Government Dental College and Hospital, Mumbai, IND; 4 Pediatric Dentistry, Pacific Dental College and Hospital, Udaipur, IND

**Keywords:** apical extrusion, debris, inoculation, irrigation, microorganism

## Abstract

Background

The present study aimed to assess the efficacy of a heat-treated, FlexiCON nickel-titanium (Ni-Ti) rotary instrumentation system and compare it with existing commonly used instrumentation systems (hand file, ProTaper Universal, and WaveOne) using the EndoVac irrigation system.

Methodology

A total of 210 single-rooted, human permanent anterior teeth were equally divided into four groups of 50 teeth each (Group I for step-back, group II for ProTaper rotary, Group III for WaveOne reciprocating, and Group IV for FlexiCON rotary instrumentation system), and 10 teeth were used as controls. Canals were irrigated with EndoVac irrigation in each group. Extruded debris, irrigating solution, and *Enterococcus faecalis* were quantified and statistically analyzed.

Results

Group IV exhibited the least amount of debris, irrigating solution, and microorganisms than other groups, while Group I presented the most. FlexiCON with EndoVac irrigation demonstrated the least amount of microbe extrusion (14 colony-forming units (CFUs)) among the four instrumentation systems, whereas step-back instrumentation with EndoVac irrigation demonstrated the most (39 CFUs). The control group showed no debris, irrigating solution, or microorganisms.

Conclusions

FlexiCON Ni-Ti rotary instrumentation showed the least debris, irrigating solution, and bacterial extrusion compared with hand, ProTaper Universal, and WaveOne reciprocating instrumentation systems when EndoVac irrigation methods were used.

## Introduction

Root canal treatment involves biomechanical preparation of the canals to eliminate all bacteria and necrotic pulp debris followed by filling with an inert material to seal the exposed tubules. The procedure aims to eliminate pain and discomfort associated with irritation of the pulp and periodontal tissues. However, the treatment is not free of complications. Periapical extrusion of irrigants or debris during or following root canal treatment is a major concern to the operator as it causes severe pain, swelling, and inflammation to the periapical apparatus. Microorganisms, their endotoxins, and, occasionally, irrigating fluid are frequently transported into periapical tissues through the apex of the dentin chips that contain the residual pulpal tissue. During the cleaning and shaping of the root canal system, any type of physical or chemical injury to the periradicular tissue can result in the degranulation of mast cells, releasing histamine into the periradicular tissue as a consequence [1]. It may encounter flare-ups and immunological reactions in some cases.

Periapical expulsion has a major role in the outcome of endodontic therapy; hence, it should either be reduced or eliminated to ensure success [2]. Although the quality (e.g., the virulence of the microbes) of an extrusion cannot be controlled, the operator can reduce its quantity (e.g., the number of microbes) with the proper instrumentation and irrigation method to avoid the above complications [2,3]. Different endodontic instruments were undertaken to minimize or prevent apical expulsion during endodontic instrumentation in the past [3-6]. Still, the elimination of the apical expulsion is unresolved.

Heat-treated files known as FlexiCON nickel-titanium (Ni-Ti) files are produced and distributed by US Endodontics (Johnson City, Tennessee, USA). These files, according to the manufacturer, offer superior cyclic fatigue resistance, shape memory, and less risk of canal zipping. They are also incredibly flexible. Because of its special parabolic cross-section, the FlexiCON Ni-Ti rotary system favors less extrusion periapically by allowing debris to exit coronally [7]. EndoVac irrigation is a novel system based on a negative-pressure technique and showed less periapical expulsion. Previous studies conducting microbial and scanning electron microscopy analysis on the effect of the EndoVac irrigation system have demonstrated it to be more effective in removing the smear layer and remnant microbes in the canal compared to conventional irrigation systems [8,9]. However, all earlier studies have assessed the ability of EndoVac when the canals are prepared with conventional rotary ProTaper files. In the context of modern endodontics, it is essential to study the irrigation systems in relation to heat-treated Ni-Ti files.

Therefore, the present in vitro study aimed to measure the amount of periapically extruded debris, irrigating solution, and microorganisms following the root canal instrumentation by heat-treated FlexiCON Ni-Ti rotary system along with EndoVac in comparison with the conventional existing instrumentation systems. This study is the first of its kind to assess the efficacy of an EndoVac irrigation system when used with a heated Ni-Ti rotary system.

## Materials and methods

The present in vitro study was conducted in accordance with the Declaration of Helsinki guidelines. The ethical approval for the study was obtained from Nair Hospital Dental College (approval number: EC-PhD-04/CONS-01/2020; dated 10/12/2020). The sample size was derived using the formula n = Z^2^ × S^2^/d^2^, where n is the calculated sample size, Z represents z-statistics of the desired level of confidence, S is the population standard deviation, and d is half the width of the desired interval. For a 95% confidence interval, the minimum sample size of 50 per group was calculated. Thus, a total of 210 human, permanent, single-rooted teeth with fully developed apices and patent oval canals were chosen. Informed consent was obtained from the patients before utilizing their teeth in the present study. Teeth with an apical curvature of 0-15° using the standard Schneider’s approach and an apical diameter that verified number #15 K-files were included in this protocol [10]. Teeth excluded from the study included teeth having cracks, caries, crown and root resorption, canal calcification, prosthetic crowns and endodontic posts, and teeth that had already undergone endodontic treatment.

All chosen teeth were cut from the coronal end to a standard length of 19 mm to preserve sample equivalency. As the cutting was performed from the incisal edge, the remainder of the coronal section of the tooth including the pulp chamber and pulp canal was kept intact and used as a reservoir for the irrigating fluid. An ideal endodontic access preparation was performed with a number 2 round carbide bur with a high-speed rotary handpiece (FG number 2 carbide bur). The remaining pulpal tissue was extirpated using a fine barbed broach (Dentsply Maillefer, Ballaigus, Switzerland). The working length of the canals was set 1 mm less than the file penetration length, or at the point at the apex where the tip of the number #10 K-file (Mani INC., Tochigi, Japan) was barely visible.

For the instrumentation of the canals in the current study, the following methods were used: step-back (K-files and H-files, Mani INC., Tochigi, Japan), ProTaper Universal rotary (Dentsply Maillefer, Switzerland), WaveOne single file (Dentsply Maillefer, Ballaigues, Switzerland) reciprocating, and FlexiCON™ rotary systems (Edge Endo, Johnson City, Tennessee, USA).

Inoculation of *Enterococcus faecalis* biofilm

After obtaining and subculturing the *Enterococcus faecalis* strain ATCC 29212, the medium was incubated for 24 hours at 37°C in brain-heart infusion agar (Himedia Laboratories Pvt. Ltd., Mumbai, India). Freshly subcultured pure *E. faecalis* was used to prepare the bacterial suspension in brain-heart infusion broth (Himedia Laboratories Pvt. Ltd., Mumbai, India). Using a sterile micropipette (high-precision pipettes), 1 mL of bacterial suspension (final concentration of approximately 1.5 × 10^8^) was transferred to the root canal access cavities after introducing bacteria into the canals along their whole length using 10 K-files. The access cavity of each sample was sealed by Cavit™ temporary filling material (3M ESPE, Dental Goods, USA). Every sample was kept in a humid environment at 37°C for 14 days, and on the first, fourth, seventh, and tenth days, re-inoculation was performed every 72 hours using fresh culture.

Test model apparatus

The methodology of the present study followed that of earlier studies measuring extruded debris after root canal instrumentation [11,12]. The debris, irrigating fluid, and microbe (*E. faecalis* ATCC 29212) that was extruded apically during instrumentation were all collected in 2 mL plastic Eppendorf tubes. The mean weight of the empty Eppendorf tube was obtained by weighing it three times using a 10-5 g precision analytic microbalance (Shimadzu, Uni-block electronic balance AUW220D, Kyoto, Japan). This was recorded as the pre-instrumental weight.

An extra lid from another Eppendorf was cut and its center was punched out to secure the specimen tooth in an experimental Eppendorf tube. To ensure a fluid-tight seal of the fitted tooth in the Eppendorf tube lid, cyanoacrylate gel was applied. To balance internal and exterior pressure and facilitate the ejection of debris, irrigating fluid, and microorganisms during instrumentation, a 26-gauge needle was placed laterally of the tooth via the lid.

This experiment used a glass vial with a capacity of 30 mL to contain and stabilize the Eppendorf tube while it was being instrumented. The rubber cork of the vial was sliced to make room for the Eppendorf tube. To prevent bias during canal instrumentation, a rubber dam sheet was placed on the installed tooth to conceal the Eppendorf tube from the operator. During a 15-hour cycle, the complete model system, including all of the Eppendorf was sterilized in ethylene oxide gas using a semi-automatic sterilizer (KANC 125, Kausthub Enterprises, Mumbai).

Evaluation of root canal bacteria

To identify the production of biofilm, samples were obtained from each canal before cleaning and shaping. If the bacterial count of the root canal was less than 1.5 × 10^8^ colony-forming units (CFUs)/mm, the incubation procedure was repeated.

Irrigating procedures

**Figure 1:**
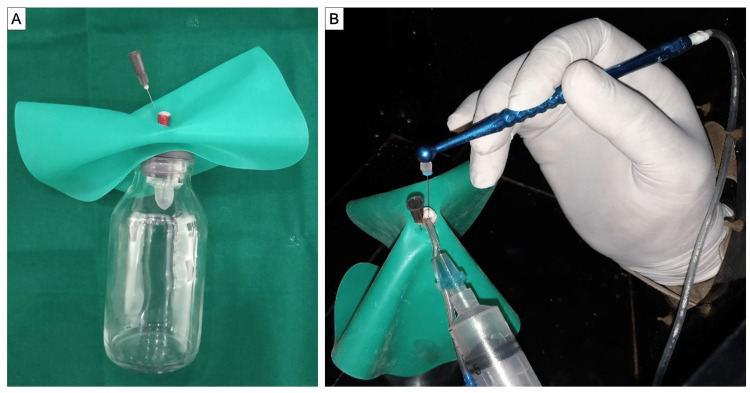
(A) Study model apparatus. (B) EndoVac irrigation system microcannula and master delivery tip in position.

Of 210 teeth, 10 were kept as control teeth which were irrigated without instrumentation. The remaining 200 teeth were randomly divided into four different groups. To eliminate bacterial contamination from the air, canal instrumentation was performed in a Class I laminar airflow cabinet. The EndoVac irrigation system was used to irrigate every tooth (Figure 1). The tubing system, macrocannula, microcannula, and master delivery tip are the four components that make up the EndoVac system. EndoVac irrigation was performed following the manufacturer’s instructions using an apical negative pressure.

Instrumentation

Every tooth was assigned into one of the four groups and instrumented using step-back, ProTaper, WaveOne, and FlexiCON instrumentation techniques. During the instrumentation process, normal saline was utilized for irrigation, and any material that was released from the apical foramen of each sample was collected in the corresponding Eppendorf tube. Step-back instrumentation was performed with K-files, and H-files were alternatively used for canal preparation. The taper of these files was 2% constant taper. Apical preparation in these was fixed to number #25 K-file and step-back till number #40 K-file.

The ProTaper Universal rotary system has a convex, triangular cross-section and a non-cutting tip. There is a progressive and variable taper on these Ni-Ti rotary files. The instrumentation was set up as follows: S1, Sx, S2, F1, and F2. File F2 has a number #25 tip diameter and the system operates in a crown-down fashion. The WaveOne reciprocating files are manufactured with M-wire Ni-Ti alloy, made up of 508 nitinol, under specific tensions and heat treatments at various temperatures. WaveOne primary (number #25) reciprocating files were used as per the manufacturer’s instructions.

Heat-treated files known as Annealed, Fire-Wire, and FlexiCON Ni-Ti files are produced and distributed by US Endodontics (Johnson City, Tennessee, USA). The file system involved negotiating files N1 17.04, N2 17.06, and N3 20.04 and finishing files C1 20.06 and C2 25.06. These files, according to the manufacturer, offer superior cyclic fatigue resistance, no form memory, and no canal transit. They are also incredibly flexible. Because of its special parabolic cross-section, the FlexiCON Ni-Ti rotary system favors less extrusion periapically by allowing debris to exit coronally. Following the completion of the root canal instrumentation, the tooth, Eppendorf tube, and needle were separated. The postoperative weight of each tube was noted. The weight of the debris and irrigating fluid was obtained collectively by deducting the preoperative Eppendorf weight from the postoperative weight.

Evaluation of apically extruded bacteria

**Figure 2:**
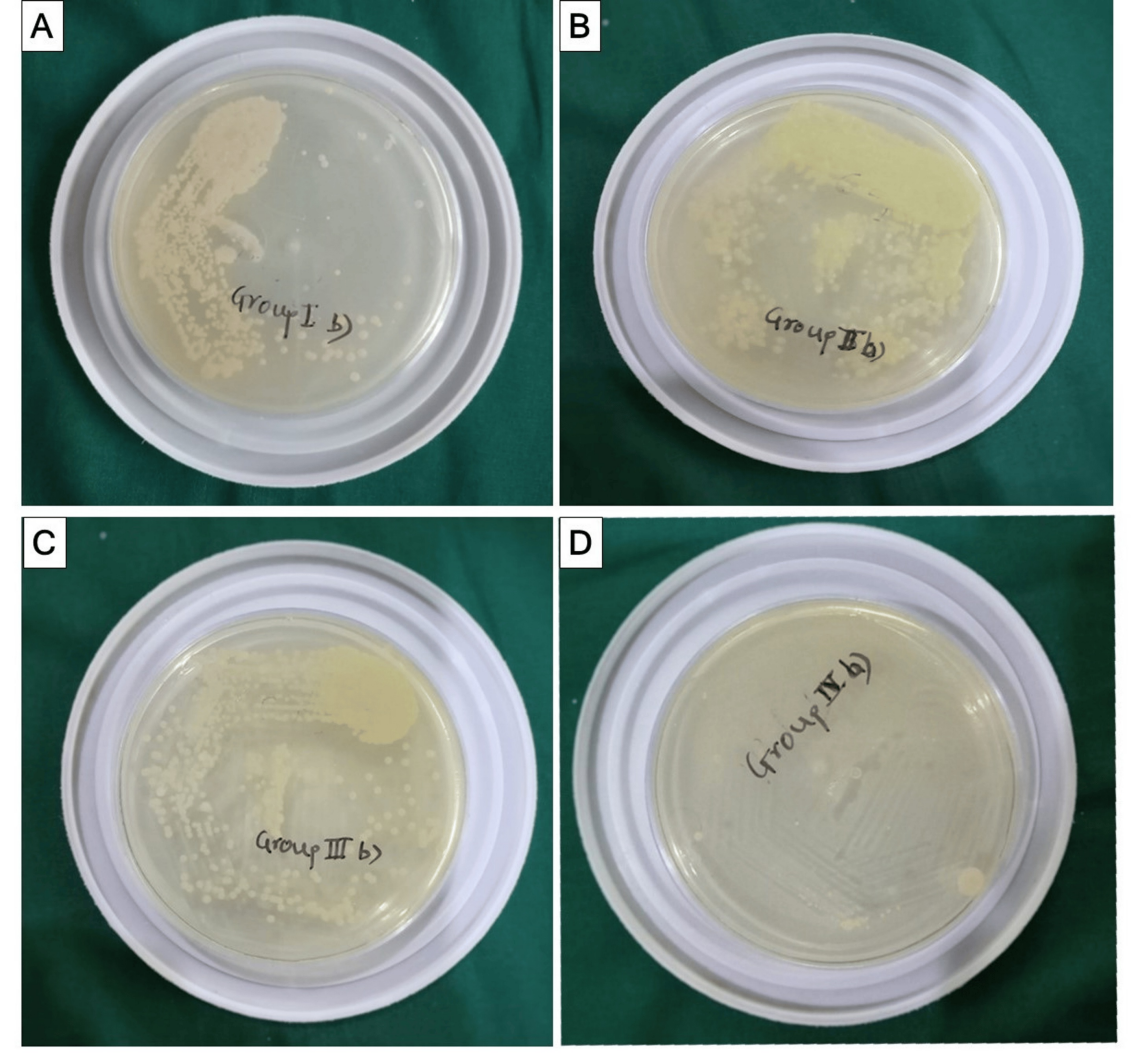
Incubation in agar for microbial counts of samples belonging to (A) Group I (manual instrumentation), (B) Group II (ProTaper instrumentation), (C) Group III (WaveOne instrumentation), (D) Group IV (FlexiCON instrumentation).

Next, 100 µL of regular saline solution was added to each Eppendorf tube, and the mixture was swirled to create a uniform mixture. The turbid saline solution of every Eppendorf tube was cultivated. De Man, Rogosa, and Sharpe agar plates were used to count the CFUs of bacteria (Figure 2), and the results were reported as the number of CFUs/mm.

Weight measurement of apically expelled dry debris

The saline solution was then allowed to evaporate by keeping the Eppendorf tube in an oven set at a steady 60°C for 30 days. For every collecting assembly, three weight measurements were made in a row, and the mean value was noted. This was referred to as the Eppendorf tube final collection assembly weight. By deducting the weight of the pre-instrumented empty Eppendorf from the total weight of the collection Eppendorf, the weight of the extruded debris was calculated. The weight of the irrigating solution was determined by deducting the weight of the extruded dry debris from the post-instrumented collection Eppendorf weight (debris + irrigating fluid) once the dry debris weight was obtained. The amount of irrigating solution was converted into milliliters from weight.

Statistical analysis

All information about the weights of the debris, the milliliter of the irrigating solution, and microbe colonies was gathered and statistically analyzed. Data analysis was done using SPSS Statistics version 20.0 (IBM Corp., Armonk, NY, USA). Descriptive and inferential statistical examinations were performed. Results of continuous measurements were presented on mean ± standard deviation (SD). The level of significance was fixed at p-values of 0.05, and any value less than or equal to 0.05 was considered to be statistically significant. Following the results of the normality test (Kolmogorov-Smirnov and Shapiro-Wilk test), it was concluded that part of the data did not follow the normal distribution; hence, non-parametric tests were applied. The significance in multiple groups was determined using the analysis of variance test and pair-wise significance within groups was determined using Tukey’s honest significant difference post-hoc test.

## Results

Apical extrusion of debris

Among the instruments, FlexiCON with EndoVac irrigation demonstrated the lowest debris expulsion (0.00458040 ± 0.010529657 g) while step-back instrumentation with EndoVac irrigation exhibited the maximum (0.02524664 ± 0.014035576 g) (Table 1). From least to most, the sequence was as follows: FlexiCON < WaveOne < ProTaper < step-back instrumentation. A statistically significant difference (p < 0.05) was observed in the amount of extruded debris across different pairs of groups (Table 2).

**Table 1:** Comparison of the apical extrusion of debris for different file systems with EndoVac irrigation using analysis of variance. *: p < 0.05: significant; **: p < 0.001: highly significant.

Group	N	Mean	Standard deviation	F-value	P-value
Step-back	50	0.02524664	0.014035576	110.419	<0.001**
ProTaper	50	0.01754380	0.008636009		
WaveOne	50	0.00544780	0.008082230		
FlexiCON	50	0.00458040	0.010529657		
Total	200	0.01320466	0.013609807		

**Table 2:** Individual pairwise comparison of the apical extrusion of debris for different file systems with EndoVac irrigation using Tukey’s post-hoc analysis. *: p < 0.05: significant; **: p < 0.001: highly significant.

	Step-back	ProTaper	WaveOne	FlexiCON
Step-back	-	0.006*	<0.001***	<0.001**
ProTaper	0.006*	-	<0.001**	<0.001**
WaveOne	<0.001**	<0.001**	-	0.021*
FlexiCON	<0.001**	<0.001**	0.021*	-

Apical extrusion of irrigant

FlexiCON with EndoVac irrigation demonstrated the least amount of irrigating solution extrusion (0.00137922 ± 0.002841851 mL) among the four instrumentation systems, whereas step-back instrumentation with EndoVac irrigation demonstrated the most (0.02147677 ± 0.007863551 mL). The order of sequence from minimum to maximum was FlexiCON < ProTaper < WaveOne < step-back instrumentation (Table 3). A statistically significant difference (p < 0.05) was observed in the amount of extruded irrigating solution across different pairs of groups (Table 4).

**Table 3:** Comparison of the apical extrusion of irrigating solutions for different file systems with EndoVac irrigation using analysis of variance. *: p < 0.05: significant; **: p < 0.001: highly significant.

Group	N	Mean	Standard deviation	F-value	P-value
Step-back	50	0.02147677	0.007863551	133.134	<0.001**
ProTaper	50	0.01519676	0.005360228		
WaveOne	50	0.01032425	0.052066620		
FlexiCON	50	0.00137922	0.002841851		
Total	200	0.01209425	0.027312574		

**Table 4:** Individual pairwise comparison of the apical extrusion of irrigating solutions for different file systems with EndoVac irrigation using Tukey’s post-hoc analysis. *: p < 0.05: significant; **: p < 0.001: highly significant.

	Step-back	ProTaper	WaveOne	FlexiCON
Step-back	-	<0.001**	<0.001**	<0.001**
ProTaper	<0.001**	-	<0.001**	<0.001**
WaveOne	<0.001**	<0.001**	-	0.012*
FlexiCON	<0.001**	<0.001**	0.012*	-

Apical extrusion of microorganisms

FlexiCON with EndoVac irrigation demonstrated the least amount of microbe extrusion (14 CFUs) among the four instrumentation systems, whereas step-back instrumentation with EndoVac irrigation demonstrated the most (39 CFUs). The order of sequence from minimum to maximum was FlexiCON < WaveOne < ProTaper < step-back instrumentation (Table 5). A statistically significant difference (p < 0.05) was observed in the amount of microbe extrusion across different pairs of groups (Table 6).

**Table 5:** Comparison of the colony forming units for different file systems with EndoVac irrigation using analysis of variance. *: p < 0.05: significant; **: p < 0.001: highly significant.

Group	N	Mean	Standard deviation	F-value	P-value
Step-back	50	39.590	5.7876	337.325	<0.001**
ProTaper	50	24.560	2.3940		
WaveOne	50	21.610	3.8323		
FlexiCON	50	14.970	3.2126		
Total	200	25.183	9.8732		

**Table 6:** Comparison of colony forming units for different file systems with Endovac irrigation using Tukey’s post-hoc analysis. *: p < 0.05: significant; **: p < 0.001: highly significant.

	Step-back	ProTaper	WaveOne	FlexiCON
Step-back	-	<0.001**	<0.001**	<0.001**
ProTaper	<0.001**	-	0.002*	<0.001**
WaveOne	<0.001**	0.002*	-	<0.001**
FlexiCON	<0.001**	<0.001**	<0.001**	-

The control group showed no debris and irrigating solution or the growth of microorganisms in the 10 Eppendorf tubes as these teeth were neither instrumented nor irrigated. There was also no growth of microbes observed while culturing the saline solution from Eppendorf tubes from the control group.

## Discussion

The goal of the present study was to assess as well as collate and contrast different conventionally used instrumentation systems such as step-back (manual instrumentation), ProTaper (commonly used rotary), WaveOne (reciprocating), and the recently introduced FlexiCON rotary instrumentation systems for apical extrusion of debris, irrigating solution, and microorganisms using the EndoVac irrigation technique. Except for the control group, all groups demonstrated apical extrusion of debris, irrigating solution, and microorganisms. Thus, the null hypothesis was rejected. Teeth from the control groups were neither instrumented nor irrigated and showed no expulsion, whereas all experimental groups showed remarkable periapical expulsion. Hence, the cleaning and shaping procedure produced periapical expulsion.

The current study examined single-rooted anterior teeth, mature apices, a patent single canal, and canal curvature not exceeding 15° using the standard Schneider’s approach to standardize the study settings [10]. Every tooth was decoronated to a consistent working length of 19 mm, with the apical size corresponding to the number 15 K-file. This ensured that the number of microbes, irrigating solution, and apical extrusion were all created by the root canal preparation and not by other factors. Teeth in this investigation were kept in sodium hypochlorite for a full day after pulpal extraction to ensure that no pulpal remnants were left within the root canal.

To eliminate the bias of dripping irrigating solution, debris, and microbes other than the apical foramen, every tooth was painted with nail varnish except at the apical foramen. The protocol of the present study model was comparable to the apparatus in the study by Myers and Montgomery or the quantification of debris, irrigating solution, and microorganisms [13]. As the purpose of the present study was to quantify bacteria, normal saline was used as an irrigant instead of usual irrigants such as sodium hypochlorite to prevent the killing of living microorganisms and, subsequently, the incorrect weight measurement of crystallization of sodium hypochlorite [14]. To eliminate variations in the weight of the expelled debris, 10 mL of normal saline was uniformly utilized for irrigation in each sample during instrumentation.

The gram-positive facultative anaerobic bacteria *E. faecalis* has been frequently found in infected root canal cases and was thus assessed in the present study [15-17]. The sample tooth was selected for instrumentation only when the growth of *E. faecalis* reached 10^8^ and above as such numbers of microbes are characteristically found in infected teeth [18]. As our study was limited to the quantification of bacterial expulsion and not related to the disinfection of root canals, 24-hour subculture was sufficient time for assessment [19-21].

To stabilize the teeth during instrumentation, the tooth was suspended in the Eppendorf tube and held in the glass vial. A rubber dam was placed over the teeth in accordance with their dimensions, and cyanoacrylate gel was applied to guarantee a fluid-tight seal. By doing this, the bias of collecting debris, irrigating fluid, and microorganisms that come exclusively from the apical foramen was confirmed. This study quantified the amount of debris, irrigating solution, and CFUs of the microorganism. The tooth type, canal size and curvature, instrument size and type, instrumentation technique and endpoint, apical preparation limit, irrigation solution, and irrigation delivery system are some of the factors that affect the apical expulsion of debris, irrigating solution, and microorganisms.

Root canal preparation (various file geometry, rotating or reciprocating movements) and irrigation (type and delivery technique) are the main causes of these periapical expulsions [22]. According to a recent comprehensive review and meta-analysis, movement type and instrument design have a greater impact on inflammatory reactions caused by debris ejection at the apical end than file types [23]. As a result, this study compared various instrumentation movement and design techniques, including reciprocating (WaveOne system), rotary (ProTaper Universal rotary system and FlexiCON (heat-treated Ni-Ti) rotary system), and hand instrumentation (K- and H-files step-back technique) with EndoVac irrigation methods.

The WaveOne reciprocating system works on the reciprocating action and simulates but reverses the balanced force technique, as theorized by Roane and Sabala [24]. Reciprocating motion delivers the advantage of conserving complex canal anatomies [25]. These files have an apical tip size of number #25 and an apical taper of 8%, which gets smaller toward the coronal end [26].

To properly irrigate the root canal system, it has been suggested that root canals be flushed with an EndoVac irrigation system, as per manufacturer instrumentation. Except for the control group, all experimental groups displayed apical extrusion of debris, irrigating solution, and microorganisms. The current investigation found that debris, irrigating solution, and microbes were expelled more from step-back instrumentation than from engine-driven (rotary and reciprocating) instrumentation. Hand files showed more expulsion because they made more contact with the dentin than rotary file designs, which increased the taper lock and screw-in effect [4,5].

When ProTaper Universal rotary instrumentation was compared with WaveOne reciprocating using EndoVac irrigation, our study showed more expulsion with ProTaper Universal than with WaveOne reciprocating file. We propose that the ProTaper Universal rotary has a convex, triangular cross-section, changing the helical angle and pitch over the cutting blade and non-cutting, modified guiding tip. Shaping instruments have a larger percentage of taper over the cutting length [6]. The reason for higher debris expulsion with ProTaper Universal could be larger apical taper results in more aggressive preparation of the root canal, which could lead to larger apical debris expulsion.

Western et al. reported in a meta-analysis that WaveOne reciprocating instrumentation mimics the balanced force kinematics, which is also known for pressure-less movement, pushing less material periapically [27]. The other factors such as instrument design, improved alloy, fewer instruments, high cutting ability, and reciprocation kinematics of the WaveOne system could have improved control of apically extruded debris.

The present study observed that FlexiCON rotary instrumentation showed minimum apical extrusion of debris, irrigating solution, and microbes than ProTaper rotary instrumentation with EndoVac irrigation. The recently introduced FlexiCON rotary, heated Ni-Ti instrumentation system showed minimum expulsion of debris. This can be explained as heated Ni-Ti files have superior flexibility and resistance to cyclic fatigue and show a less aggressive cutting tendency compared with the ProTaper system [28]. This could have resulted in less apical extrusion of debris and irrigating solution. The unique parabolic cross-section of FlexiCON rotary files pushes debris coronally, resulting in less apical extrusion compared with the convex-triangular cross-section of ProTaper files.

No comparative study has been reported in the literature until now with the EndoVac irrigation method using the above instrumentation techniques. The current study revealed that FlexiCON instrumentation showed lower debris, irrigating solution, and microorganisms than WaveOne instrumentation. FlexiCON is a heat-treated Ni-Ti system that has a thinner file diameter compared with the WaveOne system and pushes debris coronally, resulting in less debris extrusion than WaveOne. The unique parabolic cross-section of FlexiCON files tends to move debris coronally compared with the modified convex, triangular, cross-section of WaveOne reciprocating files.

Conclusively, it was observed that FlexiCON Ni-Ti rotary instrumentation showed the least debris, irrigating solution, and bacterial extrusion compared with step-back (hand), ProTaper Universal rotary, and WaveOne reciprocating instrumentation systems when conventional needle and EndoVac irrigation methods were used. A possible interpretation of such an outcome may be that heat-treated Ni-Ti FlexiCON files have unique parabolic cross-sections, better flexibility, and resistance to cyclic fatigue than other files resulting in less aggressive cutting efficiency which could lead to less expulsion. Step-back instrumentation showed maximum extrusion of debris irrespective of the irrigation method used.

The limitation of this experimental apparatus is the lack of back pressure of periapical tissue as a natural barrier for periapical extrusion. The use of floral foam has been proposed by some researchers to imitate the back pressure of periapical tissue but there are many disadvantages to absorption of irrigant and debris. Hence, back pressure like periapical tissue was not attempted in the current study. Still, more studies are needed to effectively compare EndoVac irrigation with step-back, ProTaper, WaveOne, and FlexiCON. Another uncertain factor is that if the least apical extrusion of microbes extruded by certain instrumentation and if that strain is more virulent, then periapical reaction in those patients will be severe compared with larger debris expulsion with less virulent microbes. Although the EndoVac irrigation system is considered effective in comparison to other systems, it is not being practiced often in routine clinical practice. Future studies comparing the EndoVac system to other irrigating systems are warranted to aid clinicians in adopting suitable methods in their routine practice.

## Conclusions

FlexiCON Ni-Ti rotary instrumentation showed the least debris, irrigating solution, and bacterial extrusion compared with hand, ProTaper Universal rotary, and WaveOne reciprocating instrumentation systems when the EndoVac irrigation system was used. The findings of the present study would aid clinicians in the decision-making process for the selection of files when apical extrusion is crucial.
